# Association between joint physical activity and healthy dietary patterns and hypertension in US adults: cross-sectional NHANES study

**DOI:** 10.1186/s12889-024-18346-8

**Published:** 2024-03-19

**Authors:** Yanzhou Zhu, Zhigang Wang

**Affiliations:** Department of Geriatrics, Mindong Hospital Affiliated to Fujian Medical University, Ningde, Fujian 355000 China

**Keywords:** Healthy diet index, Physical activity, Hypertension, NHANES, Association

## Abstract

**Background:**

Lack of physical activity (PA), poor dietary habits, or other unhealthy lifestyle behaviors are potential modifiable risk factors for hypertension. It has been sufficiently demonstrated in previous studies that physical activity or healthy dietary patterns can reduce the risk of hypertension. However, no research focused on the joint effects of PA and healthy dietary patterns on hypertension in a representative sample of adults.

**Methods:**

We used data collected from the 2007–2018 National Health and Nutrition Examination Survey. Healthy dietary patterns were assessed with the Healthy Eating Index 2015 (HEI-2015), and PA was measured using the metabolic equivalent minutes per week reported in questionnaires. We created four lifestyle categories based on the HEI-2015 and PA: (1) unhealthy diet and physically inactive (less than recommended PA), (2) healthy diet but physically inactive, (3) unhealthy diet but physically active (recommended PA), (4) healthy diet and physically active. Logistic regression was used to evaluate the association between joint PA and HEI-2015 and hypertension.

**Results:**

A total of 24,453 participants were enrolled in the study. Compared with unhealthy diet and physically inactive individuals, only healthy diet and physically active participants (adjusted odds ratio [AOR]: 0.77, 95% CI 0.65–0.9) were negatively associated with hypertension, while healthy diet but physically inactive participants (AOR: 0.89, 95% CI 0.76–1.03) and unhealthy diet but physically active participants (AOR: 0.9, 95% CI 0.76–1.06) were not associated with hypertension.

**Conclusion:**

In a representative sample of US adults, our findings suggest that individuals with recommended PA and healthy dietary patterns have a lower risk of hypertension than those with an unhealthy diet or less than recommended PA. Healthy eating habits and regular PA are potential preventive precautions against hypertension.

**Supplementary Information:**

The online version contains supplementary material available at 10.1186/s12889-024-18346-8.

## Introduction

Hypertension is a global health problem with a leading cause of cardiovascular disease and premature death, which affects about 1 billion adults worldwide [1]. By 2030, 41% of US adults are expected to be diagnosed with hypertension [2]. Hypertension may be caused by a complex combination of risk factors such as a high-sodium and low-potassium diet, sedentariness, smoking, alcoholism, and genetic factors [3, 4]. As hypertension is a chronically progressive disease, some risk factors can be controlled to prevent its development and progression [5].

Previous studies have reported a strong association between diet quality and PA on hypertension [6–8]. Chronic lack of physical activity (PA) is one of the risk factors for hypertension [9]. Moderate PA may contribute to lower blood pressure and improve cardiovascular health [10, 11]. PA is beneficial in enhancing heart function, inhibiting insulin resistance and inflammation in the body, promoting vasodilation, and lowering blood resistance [12, 13]. Additionally, the reduction in weight, anxiety, and depression that comes with PA can also be helpful to reduce blood pressure [14, 15]. Also, diet is strongly associated with hypertension, and a large number of studies have emphasized the importance of specific dietary patterns (e.g., the DASH diet [Dietary Approaches to Hypertension Control] and the Mediterranean diet) in controlling hypertensio n[16–18]. Healthy Eating Index 2015(HEI-2015) was developedbased on the Dietary Guidelines for Americans to assess dietary quality. HEI-2015 measures multiple aspects of diet, which provides a comprehensive assessment that can help people to form a healthy habit [19]. It has been proven that strict adherence to HEI-2015 helps aid blood pressure control in hypertensive patients [20].

Previous studies have provided sufficient evidence for the individual impact of PA or healthy dietary patterns in reducing the risk of hypertension. However, there has been no research on the joint effects of PA and healthy dietary patterns on hypertension in a representative sample of adults. To compensate for these limitations, our study used National Health and Nutrition Examination Survey (NHANES) data to explore the association between Joint PA and HEI-2015 and hypertension.

## Materials and methods

### Study population

NHANES uses a complex, multi-stage probability sampling design to select the representative, noninstitutionalized population in the US (https://www.cdc.gov/nchs/nhanes/index.htm). All participants provided written informed consent. The survey protocol was approved by the Research Ethics Review Board of the National Center for Health Statistics and the Centers for Disease Control and Prevention [21].

**Fig. 1:**
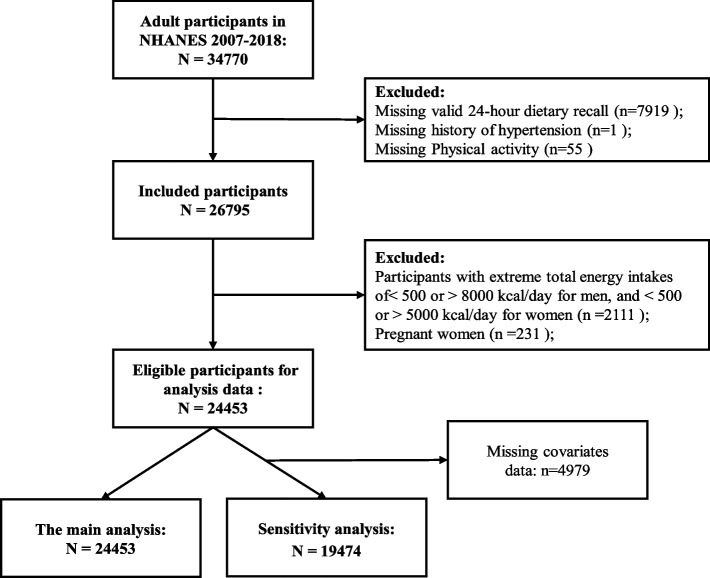
Flow diagram of inclusion criteria and exclusion criteria. Abbreviations NHANES, National Health and Nutrition Examination Survey

34,770 participants were aged 20 years or more. In the current study, we excluded individuals who missed valid 24-h dietary recall (*n* = 7,919); history of hypertension (*n* = 1); data about PA (*n* = 55). participants who had extreme total energy intakes of < 500 or > 5000 kcal/day for women, and < 500 or > 8000 kcal/day for men (*n* = 2,111) [22], and pregnant women (*n* = 231). 24,453 participants were included in the main analysis. After removing 4,979 participants with missing covariates, 19,474 participants were included in the sensitivity study (Fig. 1).

### Hypertension

A standardized blood pressure measurement protocol recommended by the American Heart Association was used between 2007 and 2018. Trained clinicians measured blood pressure using a mercury sphygmomanometer and an appropriately sized blood pressure cuff. Blood pressure was measured after 5 min of sitting still and three blood pressure readings were taken at 30-s intervals. The average of the three measurements was used to define systolic and diastolic levels. Quality control measures included quarterly recertification and retraining of clinicians as needed and annual retraining of all clinicians. Hypertension was defined as systolic blood pressure ≥ 140 mm Hg or diastolic blood pressure ≥ 90 mmHg, self-reported history of hypertension, or use of antihypertensive medications [23, 24].

### Healthy eating index 2015

Dietary intake data were obtained from two 24-h recall interviews with NHANES, conducted by a professional dietary interviewer. The first interview was conducted face-to-face, and the second interview was conducted by telephone after 3–10 days, where participants were asked to recall the types and amounts of food and beverages consumed in the past 24 h, and dietary intake was estimated using the average of the two 24-h recall data [25]. Energy and nutrient intakes for all foods were calculated using the Food and Nutrition Database for Dietary Studies [22]. NHANES Individual Food Data and Food Pattern Equivalence Database dietary data were used to construct intakes for food components for the HEI-2015.

The HEI-2015 is a tool used to assess the quality of an individual's diet. The United States Department of Agriculture Food Patterns Equivalency Database is used to convert dietary data into standardized quantities of food groups for the calculation of the HEI-2015. The HEI-2015 calculation is not based on absolute amounts of ingredients but on energy density per 1,000 kcal. It consists of 13 components, nine adequate components, including total fruits, whole fruits, total vegetables, vegetables and legumes, total protein foods, seafood and plant proteins (0–5 points each), whole grains, dairy products, and fatty acids (0–10 points each), with higher intakes scoring higher, and four moderating components, including sodium, refined grains, added sugars, and saturated fats (0–10 points each), with lower intakes, the higher the score [26]. The HEI-2015 was calculated using SAS codes [27], and higher scores indicate better overall dietary quality. This implies better compliance with DGA recommendations [26]. Participants with a two-day average score at or above the 60th percentile on the HEI were considered individuals with healthy diets (adhering to dietary guidelines or consuming healthy foods); otherwise, they were considered individuals with unhealthy diets [28].

### Physical activity

Information on PA was collected using a global PA questionnaire based on that created by the World Health Organization [29]. Participants were asked to report their PA behaviors in the last 30 days. Levels of three PA types were examined: strenuous work activity/recreational activity, moderate work activity/recreational activity, and walking/cycling activity. The number of days per week they engaged in each type of PA in a typical week was reported. and the amount of time (in minutes) spent on that type of activity during the day. The frequency and duration of these activities were used to calculate weekly metabolic equivalents (Met) estimations. NHANES provides a Met corresponding to each activity category to determine activity intensity [30].

First, Met/week was calculated by multiplying the total number of minutes per week for each activity by the NHANES-recommended MET value [31], as well as the total sum of all activities was calculated by summing over all activity categories. Secondly, respondents were categorized according to compliance with US PA guidelines (moderate intensity PA in adults should be performed for 150 min per week [equivalent to 600 Met minutes/week]). Participants were classified as physically inactive individuals (600 < Met minutes/week, less than PA recommended) and physically active individuals (≥ 600 Met minutes/week, PA recommended) [32].

### Lifestyle category

According to previous studies [33, 34], we created four lifestyle categories based on the HEI-2015 and PA: (1) unhealthy diet and physically inactive, (2) healthy diet but physically inactive, (3) unhealthy diet but physically active, (4) healthy diet and physically active.

### Covariates

Covariates included in this study include gender ( male or female), age, race/ethnicity (Non-Hispanic White, Non-Hispanic Black, Mexican American or Other Race), education levels (below high school, high school or college/above), poverty, marital (widowed or divorced or separated, never married, married or living with a partner), alcohol drinker (never, former or now), smoking status (current smoker, former smoker or never smoked), diabetes, hyperlipidemia, body mass index (BMI, calculated as weight in kilograms divided by height in meters squared), total cholesterol, high-density lipoprotein cholesterol (HDL-C).

### Statistical analysis

Data in this study were weighted using dietary interview sample weights provided by NHANES to account for the complexity of the NHANES database survey design [35]. All statistical analyses were performed using SAS 9.4 (version 9.4, SAS Institute) and R Studio software (4.2.1), and a two-sided *P* < 0.05 was considered statistically significant.

Continuous variables were described as mean and Confidence interval (95%CI), and categorical variables were expressed as proportions (%). Variance and chi-squared tests were used to analyze differences between groups [36]. To assess whether PA and HEI-2015 could modify the risk of hypertension, we calculated interactions on multiplicative and additive scales. Considering the exclusion of 4,979 participants with missing covariates, we used multiple interpolations for further analysis of 24,453 participants. Multiple interpolations were performed using Surveyimpute and MIANALYZE methods.

The association between PA and HEI-2015 and hypertension was assessed using multivariable logistic regression analyses, and the regression models were well-fitted (Supplementary Table 1) Results were expressed as odds ratio (OR) and 95% confidence interval (95% CI). Model 1 was adjusted for age, gender, and race. Model 2 was adjusted for model 1 plus education levels, poverty, marital, alcohol drinker (never, former, or now), and smoking status. And model 3 was further adjusted for model 2 plus diabetes, hyperlipidemia, BMI, total cholesterol, and HDL-C. We investigated the nonlinear association between PA and HEI-2015 and hypertension by including restricted cubic spline curves with nodes at the 10th, 50th, and 90th quartiles in the fully adjusted model. In addition, we used the data after excluding the missing values (*n* = 4979) and repeated the analysis to assess the robustness of our results.

## Results

A total of 24,453 participants were enrolled in the main analysis, including 8570 hypertension. The weighted HEI-2015 was 54.2, and the PA level was 3762.36 Met-minutes/week. The mean age was 47.73 years, of which 49.17% were female. According to the lifestyle categories, age, poverty, gender, race/ethnicity, education levels, smoking status, alcohol drinker, marital, hyperlipidemia, diabetes, BMI, and HDL-C have significant differences between groups (*P* < 0.05) (Table 1).

**Table 1:** Baseline characteristics of participants by lifestyle categories among 24,453 US adults in NHANES (2007–2018)

	**Lifestyle categories, n (weighted %)**					
**Characteristics**	**Total**	**Healthy diet and physically active**	**Healthy diet but physically inactive**	**Unhealthy diet and physically inactive**	**Unhealthy diet but physically active**	***P* value**
**Hypertension**	10,938(38.5)	3001(33.1)	2731(50.0)	2653(47.6)	2553(32.7)	< 0.001
**Age (years),** mean (95% CI)	48.2(47.6,48.7)	48.2(47.3,49.1)	56.1(55.3,56.9)	50.6(49.8,51.4)	42.6(41.9,43.2)	< 0.001
**Gender**						< 0.001
Male	12,204(50.1)	3893(49.6)	1845(36.0)	2263(44.5)	4203(61.4)	
Female	12,249(49.9)	3685(50.4)	3087(64.0)	2769(55.5)	2708(38.6)	
**Races/ethnicity**						< 0.001
Non-Hispanic white	10,513(66.8)	3257(69.4)	1899(62.6)	2198(65.2)	3159(67.2)	
Non-Hispanic black	5249(11.1)	1383(8.7)	1005(10.6)	1296(14.4)	1565(11.9)	
Mexican American	3469(8.5)	1047(7.5)	755(9.0)	717(8.5)	950(9.2)	
Other Race	5222(13.7)	1891(14.4)	1273(17.7)	821(11.9)	1237(11.8)	
**Educations Levels**						< 0.001
Below high school	5643(14.8)	1296(9.8)	1359(17.9)	1520(21.6)	1468(14.6)	
High School	5601(22.9)	1330(17.0)	1074(21.3)	1321(28.3)	1876(26.9)	
College/Above	13,184(62.2)	4946(73.3)	2491(60.8)	2185(50.0)	3562(58.5)	
**Smoking status**						< 0.001
Never	13,642(56.4)	4691(61.3)	2983(60.1)	2484(51.0)	3484(52.4)	
Former	6172(25.2)	2011(27.3)	1374(29.1)	1263(23.2)	1524(22.0)	
Now	4627(18.4)	871(11.4)	574(10.8)	1281(25.8)	1901(25.6)	
**Alcohol drinker**						< 0.001
Never	3210(10.4)	940(10.0)	926(16.9)	692(12.3)	652(9.1)	
Former	3721(12.3)	879(9.8)	901(16.6)	1033(19.0)	908(11.8)	
Now	15,480(69.9)	5210(80.2)	2633(66.5)	2822(68.7)	4815(79.1)	
**Marital**						< 0.001
Widowed or divorced or separated	5513(18.1)	1422(15.2)	1475(24.6)	1365(22.1)	1251(15.4)	
Never married	4252(18.5)	1279(18.0)	502(11.1)	779(14.9)	1692(25.2)	
Married or living with a partner	14,678(63.4)	4874(66.8)	2953(64.3)	2886(63.0)	3965(59.4)	
**Poverty,** mean (95% CI)	3.0(2.9,3.1)	3.4(3.3,3.5)	3.0(2.9,3.1)	2.7(2.6,2.8)	2.9(2.8,2.9)	< 0.001
**Hyperlipidemia**	17,274(69.2)	5164(66.4)	3748(74.9)	3783(75.3)	4579(65.5)	< 0.001
**Diabetes**	3754(11.2)	887(8.3)	1133(17.4)	1003(15.4)	731(8.4)	< 0.001
**BMI (kg/m**^**2**^**),** mean (95% CI)	29.1(28.9,29.3)	27.8(27.6,28.0)	29.8(29.5,30.2)	30.7(30.3,31.0)	29.3(29.0,29.5)	< 0.001
**Total cholesterol (mg/dl),** mean (95% CI)	194.0 (193.0,195.1)	194.6 (193.0,196.3)	195.2 (193.0,197.3)	194.5 (192.6,196.5)	192.4 (190.6,194.2)	0.1
**HDL-C (mg/dl),** mean (95% CI)	53.0(52.6,53.5)	56.2(55.5,56.9)	54.1(53.4,54.9)	50.4(49.7,51.1)	50.6(50.0,51.2)	< 0.001
**Hei-2015, (Score),** mean (95% CI)	54.1(53.7,54.6)	65.5(65.2,65.9)	63.9(63.6,64.3)	43.2(42.9,43.5)	43.2(42.9,43.5)	< 0.001
**Hei-2015 levels, (Score)**						< 0.001
< 53.5	11,943(49.9)	0(0.0)	0(0.0)	5032(100.0)	6911(100.0)	
≥ 53.5	12,510(50.1)	7578(100.0)	4932(100.0)	0(0.0)	0(0.0)	
**PA, Met-minutes/week,** mean (95% CI)	3638.4 (3503.8,3773.0)	4734.7 (4521.3,4948.2)	127.0 (117.6, 136.4)	118.0 (109.3, 126.6)	6436.7 (6146.8,6726.5)	< 0.001
**Physical activity levels, (Met-minutes/week)**						< 0.001
Inactive (< 600)	9964(35.3)	0(0.0)	4932(100.0)	5032(100.0)	0(0.0)	
Increased PA (≥ 600)	14,489(64.7)	7578(100.0)	0(0.0)	0(0.0)	6911(100.0)	

Numbers (n) in the table were unweighted

Percentages or mean (95% CI) were estimated using US population weights.Race/ethnicity was determined using preferred terminology from the National Center for Health Statistics as non-Hispanic white, non-Hispanic black, and Mexican American. Mexican–American individuals were oversampled rather than broader groups of individuals from Latin America. Other include Asian, other Hispanic, Alaskan native, and multiracial individuals

Abbreviations: *N* Number, *Met* Metabolic equivalent, *BMI*, Body mass index, *HEI* Healthy Eating Index, *PA* Physical activity

### Association between PA or HEI-2015 and hypertension

We first assessed the single effect of HEI-2015 or PA on hypertension (Table 2). In unadjusted for covariates (model 1), participants with healthy diet were negatively associated with hypertension compared to individuals with unhealthy diet (OR: 0.72, 95% CI 0.65–0.80), and this association remained even after adjusting for age, gender, race, and education levels, poverty, marital, smoking status, alcohol consumption, diabetes, BMI, fast total cholesterol, and HDL-C (model 3) (OR: 0.86, 95% CI 0.77–0.96). Similarly, there was a negative association between PA and hypertension. Physically active participants had a lower risk of developing hypertension compared to physically inactive individuals (OR: 0.88, 95% CI 0.78–0.99 in model 3).

**Table 2:** Association between joint PA and Healthy Eating Index-2015 and their combined effect and hypertension after using survey impute for multiple interpolation (*n* = 24,453)

**Hypertension**	**Model 1**		**Model 2**		**Model 3**^**a**^	
	**OR (95% CI)**	***P* value**	**OR (95% CI)**	***P* value**	**OR (95% CI)**	***P* value**
**Healthy Eating Index-2015**						
Unhealthy diet	1(ref)		1(ref)		1(ref)	
Healthy diet	0.72(0.65,0.8)	< 0.01	0.76(0.69,0.84)	< 0.01	0.86(0.77,0.96)	0.01
**Physical activity level**						
Physically inactive	1(ref)		1(ref)		1(ref)	
Physically active	0.73(0.66,0.81)	< 0.01	0.76(0.68,0.84)	< 0.01	0.88(0.78,0.99)	0.04
**Lifestyle categories**						
Unhealthy diet and physically inactive	1(ref)		1(ref)		1(ref)	
Healthy diet but physically inactive	0.79(0.69,0.91)	< 0.01	0.83(0.72,0.95)	0.04	0.89(0.76,1.03)	0.9
Unhealthy diet but physically active	0.8(0.69,0.93)	< 0.01	0.81(0.7,0.95)	< 0.01	0.9(0.76,1.06)	0.7
Healthy diet and physically active	0.56(0.49,0.65)	< 0.01	0.61(0.52,0.7)	< 0.01	0.77(0.65,0.9)	< 0.01

Model 1 was adjusted for age, gender, and race

Model 2 was adjusted for age, gender, race, and education levels, poverty, marital, smoking status, alcohol consumption

Model 3 was adjusted for the variables in model 2 plus hyperlipidemia, diabetes, BMI, fast total cholesterol, and HDL-C

^a^Model 3 at HEI-2015 adjusted for the variables in model 2 plus hyperlipidemia, diabetes, BMI, fast total cholesterol, HDL-C, and PA. Model 3 at PA level adjusted for the variables in model 2 plus hyperlipidemia, diabetes, BMI, fast total cholesterol, HDL-C, and HEI-2015

Abbreviations: *OR* Odds ratio, *CI* Confidence interval, *BMI* Body mass index, *HDL-C* High-density lipoprotein cholesterol, *PA* Physical activity

### Joint association between PA and HEI-2015 and hypertension

According to the PA and HEI-2015, we have divided the lifestyle categories into four groups (unhealthy diet and physically inactive, healthy diet but physically inactive, unhealthy diet but physically active, and healthy diet and physically active) and assessed the association of lifestyle categories with hypertension. Interestingly, after adjusting for age, gender, race, education levels, poverty, marital, smoking status, alcohol consumption, diabetes, BMI, fast total cholesterol, and HDL-C, compared to the unhealthy diet and physically inactive participants, only healthy diet and physically active individuals were negatively associated with hypertension (OR: 0.77, 95% CI 0.65–0.9), whereas no association with hypertension were observed for healthy diet but physically inactive individuals (OR: 0.89, 95% CI 0.76–1.03) and unhealthy diet but physically active individuals (OR: 0.9, 95% CI 0.76–1.06).

**Fig. 2:**
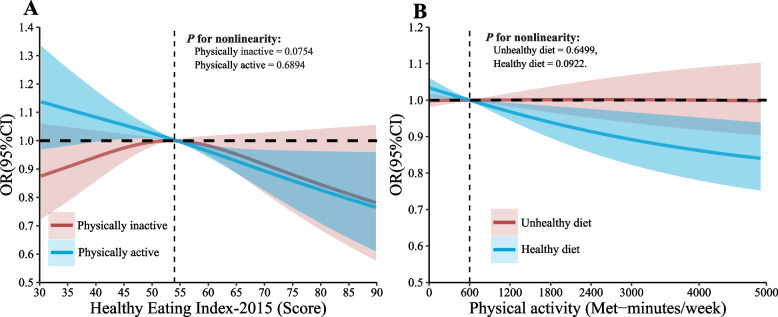
Adjusted spline curves analyze for the association of HEI-2015 (**A**) or PA (**B**) with Hypertension Among US Adults with Hypertension. Solid lines were odds ratios with 95% CI in shaded areas. Knot locations were the 10th, 50th, and 90th tertiles of PA. All models were adjusted for age, gender, race, education levels, poverty, marital, smoking status, alcohol consumption, diabetes, BMI, fast total cholesterol, and HDL-C. Abbreviations: BMI, body mass index, HDL-C, high-density lipoprotein cholesterol, HEI, Healthy Eating Index; PA, physical activity

Although the additive interaction between PA and HEI-2015 was not statistically significant (RERI =  − 0.052,95%CI − 2.149 ~ 2.044; AP =  − 0.078,95%CI − 3.333 ~ 3.177; S = 1.189, 95%CI 0.435 -3.837) (Supplementary Table 2). However, we did RCS analyses with PA (physically inactive or physically active) and HEI-2015 (healthy diet or unhealthy diet) respectively. It was found that HEI-2015 showed an inverse dose relationship with hypertension in the physically active group and PA also showed an inverse dose relationship with hypertension in the healthy diet group (Fig. 2).

### Subgroup analyses and sensitivity analyses

The main analysis of the lifestyle-hypertension association based on demographic subgroups showed similar ORs within each group after adjusting for covariates (Supplementary Table 3). Results indicated heterogeneity in the joint association between lifestyle and hypertension between Races/ethnicity. There was a statistically significant difference in the joint association between lifestyle and hypertension among non-Hispanic whites (0.56,95% CI 0.46–0.68) and Mexican-Americans (0.66, 95% CI 0.48–0.91) who were healthy diet but physically active compared to unhealthy eaters and physically inactive individuals.

To assess potential bias from missing values, we used the data after excluding the missing values (*n* = 4979) and repeated the analysis. The analysis was consistent with the results of the main analysis. After adjusting for all of the covariates, compared with the unhealthy diet and physically inactive individuals, the OR for hypertension was 0.75(95% CI, 0.63–0.89) for the healthy diet and physically active participants (Supplementary Table 4).

## Discussion

In this study, we evaluated the joint effect of PA with healthy dietary patterns on hypertension by using the NHANES database. Interestingly, the results showed that the risk of hypertension was lower among participants who followed a healthy dietary pattern and had recommended PA. Our findings provide new evidence for the association between joint healthy dietary patterns and recommended PA and a lower risk of hypertension than those with an unhealthy diet or less than recommended PA. Furthermore, our findings emphasize the combined effect of PA and HE-2105 on hypertension, and that the benefits to reduce the risk of hypertension cannot be achieved by failing to fulfill either of the above two.

PA is known as a modifiable lifestyle change associated with a variety of health outcomes. In a 12-week aerobic exercise trial, it was found that The mean decrease in systolic blood pressure by aerobic exercise was 7.1 mmHg, and the mean decrease in diastolic blood pressure was 5.1 mmHg [37]. However, most studies have focused on the benefits of recreational PA, and little research has been conducted on non-recreational PA. The NHANES assesses participants' PA intensity by collecting information on their work, transport, and recreational activities, which is more consistent with lifestyle [38]. Our study provides additional evidence that PA intensity is strongly associated with hypertension. PA can affect blood pressure through several mechanisms. Studies have shown that PA promotes cardiovascular health, reduces body weight, and improves peripheral arterial resistance [13]. which may explain the association between PA and the prevalence of hypertension [6]. In addition, physical activity may reduce stress-related psychological disorders such as anxiety and depression, further helping to control blood pressure and reduce the incidence of hypertension [14].

A large number of studies conducted in the last decades have emphasized the importance of diet in the risk of hypertension [16–18, 39]. High sodium intake may contribute to the development of hypertension, while sodium excretion, a surrogate marker of sodium intake, is also highly correlated with blood pressure levels in hypertensive patients [40]. An increase in dietary sodium content leads to an accumulation in sodium retention, which raises the venous tone and central blood volume, thus contributing to the development of hypertension [41]. A plant-based diet consists of high fiber, antioxidants, high potassium, and low saturated fat and sodium [42, 43]. A plant-based diet prevents hypertension and has beneficial effects on blood viscosity, vasodilatation, and reduction of insulin resistance [44]. In addition, with its antioxidant and anti-inflammatory properties and valuable fiber content, it can alter colonies and strains of intestinal flora and improve blood pressure by affecting the renin-angiotensin system and pressure receptors [45]. According to Lea Borgi, long-term fruit use may reduce the risk of developing hypertension [46]. A study conducted in Bangladesh found that higher fruit and vegetable intake was associated with lower annual pulse pressure and systolic blood pressure, while higher meat intake was associated with higher pulse pressure [47].

However, there is a growing emphasis in dietary studies on evaluating overall dietary patterns instead of isolated nutrients or food groups. This approach recognizes that dietary components are interconnected and consumed together[48]. By evaluating dietary patterns, one can overcome the complexity of assessing individual foods or nutrients and their interactions, thus gaining a comprehensive understanding of how overall dietary quality relates to hypertension [49, 50]. Numerous studies have conclusively shown that adopting a healthy diet is strongly correlated with reduced incidence of hypertension and lower mortality rates attributed to hypertension [5, 51, 52]. The HEI-2015 was proposed as a composite measure of dietary quality by the Dietary Guidelines for Americans (DGA), based on evidence-based recommendations from the U.S. Departments of Agriculture (USDA) and Health and Human Services (HHS). The HEI 2015 criteria have been shown to be a reliable and accurate measure of nutritional quality for Americans [48], and related studies have also demonstrated that stricter adherence can bring a preventive effect on the development of hypertension [20].

Although recommended PA and healthy diet quality are both independently associated with a reduced risk of hypertension, few studies have examined the combined effects of these lifestyle factors. Interestingly, our study found that the healthy diet and physically active participants alone were negatively associated with hypertension. Previous research has also shown that a DASH diet and exercise significantly reduces daytime ambulatory SBP compared with a DASH diet alone in a hypertensive population [53]. This is consistent with the results of this study. It is important to note that dietary patterns and PA have a strong influence on health and disease risk. Although high levels of PA may help to mitigate the negative effects of certain poor dietary habits, unhealthy eating habits may still increase the risk of developing hypertension in the long run [6, 54]. Therefore, a combination of healthy eating habits and adequate PA is the key to preventing hypertension.

The strengths of this study included a nationally representative design, information on three PA types (strenuous work activity/recreational activity, moderate work activity/recreational activity, and walking/cycling activity), and comprehensive as well as detailed dietary information. Despite this, there are limitations to our study. First, our cross-sectional study design made it difficult to determine causal associations between HEI and PA and hypertension. In the future, prospective observational and intervention studies are needed to explore how PA and dietary patterns prevent hypertension. Second, In NHANES, interviews and questionnaires were used to collect data, and data on diet, bowel, and PA were based on self-reported measurements. This may lead to inaccurate information and recall bias. In addition, PA collected by NHANES is the participant's subjective conscious report of PA, so unconscious PA may be ignored.

## Conclusion

Our study shows that recommended PA combined and healthy dietary patterns were negatively associated with the risk of hypertension in a representative sample of U.S. adults. This implies that the combination of recommended physical activity with healthy dietary patterns to prevent hypertension is a practical approach. Therefore, our findings help to identify specific lifestyles that are effective in the prevention of hypertension and provide a useful reference for policymakers to initiate relevant prevention programs and promote them to the public.

### Supplementary Information

**Supplementary Material 1.**

## Data Availability

All data used in this study is available in NHANES database. NHANES—National Health and Nutrition Examination Survey Homepage (cdc.gov).
