# Supplementary material for: Association between joint physical activity and healthy dietary patterns and hypertension in US adults: cross-sectional NHANES study
Source: BMC Public Health. 2024 Mar 19;24:855. doi: 10.1186/s12889-024-18346-8 (PMC10953194; doi:10.1186/s12889-024-18346-8)
Supplement: Supplementary file 1 — Supplementary Material 1. [file 12889_2024_18346_MOESM1_ESM.docx]

**Supplementary Online Content**

**Supplementary Table 1.** Goodness-of-fit statistics for logistic regression regression models.

**Supplementary Table 2.** Additive interaction between PA and HEI-2015.

**Supplementary Table 3.** Associations between exposure to different lifestyle categories and hypertension in stratified analyses (n=19474).

**Supplementary Table 4.** Association between PA and Healthy Eating Index-2015 and their combined effect and hypertension (n=19474).

**Supplementary Table 1.** Goodness-of-fit statistics for logistic regression regression models.

|  | **AIC** |  | **SC** |  | **-2 Log L** |  |
| --- | --- | --- | --- | --- | --- | --- |
|  | **Intercept Only** | **Intercept and Covariates** | **Intercept Only** | **Intercept and Covariates** | **Intercept Only** | **Intercept and Covariates** |
| **Healthy Eating Index-2015** |  |  |  |  |  |  |
| Model 1 | 274359866 | 222135485 | 274359883 | 222135605 | 274359864 | 222135471 |
| Model 2 | 236815867 | 191038843 | 236815884 | 191039115 | 236815865 | 191038811 |
| Model 3 | 226373347 | 172250895 | 226373364 | 172251251 | 226373345 | 172250853 |
| **Physical activity level** |  |  |  |  |  |  |
| Model 1 | 274359866 | 222295826 | 274359883 | 222295946 | 274359864 | 222295812 |
| Model 2 | 236815867 | 190906610 | 236815884 | 190906882 | 236815865 | 190906578 |
| Model 3 | 226373347 | 172219934 | 226373364 | 172220290 | 226373345 | 172219892 |
| **Lifestyle categories** |  |  |  |  |  |  |
| Model 1 | 274359866 | 221454384 | 274359883 | 221454538 | 274359864 | 221454366 |
| Model 2 | 236815867 | 190369059 | 236815884 | 190369365 | 236815865 | 190369023 |
| Model 3 | 226373347 | 172092724 | 226373364 | 172093114 | 226373345 | 172092678 |

The model was adjusted for age, sex, race, and education levels, poverty, marital, smoking status, alcohol consumption, hyperlipidemia, diabetes, BMI, fast total cholesterol, HDL-C.

The “Model Fit Statistics” table contains the AIC, the SC, and the negative of twice the Log L for the intercept-only model and the ﬁtted model. AIC and SC can be used to compare different models, and the ones with smaller values are preferred.

**Abbreviations**: OR, odds ratio, CI, confidence interval; AIC, akaike information criterion; SC, schwarz criterion; Log L, log likelihood; BMI, body mass index; HDL-C, high density lipoprotein cholesterol.

**Supplementary Table 2.** Additive interaction between PA and HEI-2015.

| **Indicators** | **Estimated value** | **95% CI** |
| --- | --- | --- |
| **RERI** | -0.057 | -0.260 - 0.145 |
| **AP** | -0.077 | -0.344 - 0.191 |
| **S** | 1.292 | 0.435 -3.837 |

The model was adjusted for age, sex, race, and education levels, poverty, marital, smoking status, alcohol consumption, hyperlipidemia, diabetes, BMI, fast total cholesterol, HDL-C.

**Abbreviations**: CI, confidence interval; RERI, relative excess risk due to interaction; AP, attributable proportion; S, synergy index; HEI, Healthy Eating Index; PA, physical activity; BMI, body mass index; HDL-C, high density lipoprotein cholesterol.

**Supplementary Table 3.** Associations between exposure to different lifestyle categories and hypertension in stratified analyses (n=19474).

| **Character** | Healthy diet but Physically inactive | Unhealthy diet but Physically active | Healthy diet and Physically active | *P* for trend | *P* for interaction |
| --- | --- | --- | --- | --- | --- |
| **Age group** |  |  |  |  | 0.52 |
| ≤65 years | 1.06(0.87,1.30) | 0.68(0.57,0.80) | 0.66(0.55,0.78) | <0.0001 |  |
| >65 years | 0.91(0.67,1.22) | 0.74(0.51,1.05) | 0.52(0.37,0.75) | <0.0001 |  |
| **Gender** |  |  |  |  | 0.29 |
| Male | 1.30(1.01,1.66) | 0.64(0.53,0.78) | 0.67(0.54,0.84) | <0.0001 |  |
| Female | 1.05(0.86,1.30) | 0.61(0.49,0.76) | 0.59(0.49,0.71) | <0.0001 |  |
| **Races/ethnicity** |  |  |  |  | 0.03 |
| Non-Hispanic white | 1.26(1.00,1.58) | 0.56(0.45,0.69) | 0.56(0.46,0.68) | <0.0001 |  |
| Non-Hispanic black | 1.16(0.92,1.47) | 0.64(0.48,0.85) | 0.90(0.70,1.17) | 0.05 |  |
| Mexican American | 0.80(0.58,1.11) | 0.76(0.54,1.09) | 0.66(0.48,0.91) | 0.02 |  |
| Other Race | 1.12(0.76,1.63) | 0.96(0.70,1.31) | 0.95(0.66,1.37) | 0.54 |  |
| **Educations Levels** |  |  |  |  | 0.19 |
| Below high school | 1.25(0.92,1.71) | 0.70(0.52,0.95) | 0.79(0.56,1.12) | 0.03 |  |
| High School | 1.29(0.95,1.76) | 0.68(0.49,0.94) | 0.80(0.58,1.11) | 0.02 |  |
| College/Above | 1.08(0.85,1.36) | 0.58(0.48,0.71) | 0.56(0.47,0.67) | <0.0001 |  |
| **Marital** |  |  |  |  | 0.49 |
| Widowed or divorced or separated | 1.20(0.88,1.62) | 0.75(0.55,1.02) | 0.71(0.52,0.96) | 0.002 |  |
| Never married | 1.20(0.79,1.80) | 0.46(0.33,0.64) | 0.54(0.38,0.75) | <0.0001 |  |
| Married or living with partner | 1.14(0.91,1.42) | 0.62(0.52,0.75) | 0.62(0.52,0.74) | <0.0001 |  |
| **BMI, (kg/m^2^)** |  |  |  |  | 0.58 |
| Healthy weight (≤25) | 1.19(0.91,1.56) | 0.68(0.57,0.81) | 0.69(0.55,0.85) | <0.0001 |  |
| Overweight (25-30) | 1.27(0.97,1.68) | 0.55(0.41,0.75) | 0.66(0.49,0.88) | <0.0001 |  |
| Obesity (≥30) | 1.11(0.82,1.51) | 0.69(0.50,0.95) | 0.80(0.58,1.09) | 0.03 |  |

Model was adjusted for age, sex, race. and education levels, poverty, marital, smoking status, alcohol consumption, hyperlipidemia, diabetes, BMI, fast total cholesterol, HDL-C.

All ORs were referenced to the unhealthy diet and physically inactive group.

**Abbreviations**: OR, odds ratio, CI, confidence interval; HEI, Healthy Eating Index; PA, physical activity; BMI, body mass index; HDL-C, high density lipoprotein cholesterol.

**Supplementary Table 4.** Association between PA and Healthy Eating Index-2015 and their combined effect and hypertension (n=19474).

| **Hypertension** | **Model 1** |  | **Model 2** |  | **Model 3*** |  |
| --- | --- | --- | --- | --- | --- | --- |
|  | **OR (95% CI)** | ***P* value** | **OR (95% CI)** | ***P* value** | **OR (95% CI)** | ***P* value** |
| **Healthy Eating Index-2015** |  |  |  |  |  |  |
| Unhealthy diet | 1(ref) |  | 1(ref) |  | 1(ref) |  |
| Healthy diet | 0.72(0.65,0.79) | <0.01 | 0.75(0.67,0.84) | <0.01 | 0.86(0.76,0.97) | 0.02 |
| **Physical activity level** |  |  |  |  |  |  |
| Physically inactive | 1(ref) |  | 1(ref) |  | 1(ref) |  |
| Physically active | 0.73(0.66,0.81) | 0.02 | 0.72(0.64,0.81) | 0.01 | 0.86(0.75,0.98) | 0.03 |
| **Lifestyle categories** |  |  |  |  |  |  |
| Unhealthy diet and physically inactive | 1(ref) |  | 1(ref) |  | 1(ref) |  |
| Healthy diet but physically inactive | 0.79(0.69,0.91) | <0.01 | 0.84(0.71,0.99) | 0.04 | 0.92(0.76,1.1) | 0.35 |
| Unhealthy diet but physically active | 0.8(0.69,0.93) | <0.01 | 0.79(0.67,0.93) | <0.01 | 0.89(0.74,1.07) | 0.2 |
| Healthy diet and physically active | 0.56(0.49,0.65) | <0.01 | 0.58(0.5,0.67) | <0.01 | 0.75(0.63,0.89) | <0.01 |

Model 1 was adjusted for age, sex, and race.

Model 2 was adjusted for age, sex, race, and education levels, poverty, marital, smoking status, alcohol consumption.

*Model 3 at HEI-2015 adjusted for the variables in model 2 plus hyperlipidemia, diabetes, BMI, fast total cholesterol, HDL-C, and PA. Model 3 at PA level adjusted for the variables in model 2 plus hyperlipidemia, diabetes, BMI, fast total cholesterol, HDL-C, and HEI-2015.

**Abbreviations**: OR, odds ratio, CI, confidence interval; PA, physical activity; BMI, body mass index; HDL-C, high-density lipoprotein cholesterol.
